# A cross-sectional study of the prevalence and correlates of tobacco Use in Chennai, Delhi, and Karachi: data from the CARRS study

**DOI:** 10.1186/s12889-015-1817-z

**Published:** 2015-05-11

**Authors:** Carla J Berg, Vamadevan S. Ajay, Mohammed K. Ali, Dimple Kondal, Hassan M. Khan, Roopa Shivashankar, Rajendra Pradeepa, Deepa Mohan, Zafar Fatmi, Muhammad M. Kadir, Nikhil Tandon, Viswanathan Mohan, KM Venkat Narayan, Dorairaj Prabhakaran

**Affiliations:** Department of Behavioral Sciences and Health Education, Emory University Rollins School of Public Health, 1518 Clifton Rd NE, 30322 Atlanta, Georgia USA; Public Health Foundation of India, Plot No 47, Sector 44, 122002 Gurgaon, Haryana India; Centre for Chronic Disease Control, Plot No 47, Sector 44, 122002 Gurgaon, Haryana India; Hubert Department of Global Health, Emory University Rollins School of Public Health, 1518 Clifton Rd NE, 30322 Atlanta, Georgia USA; Aga Khan University, Stadium Road, P.O. Box 3500, 74800 Karachi, Pakistan; Madras Diabetes Research Foundation, No 4, Conran Smith Road, Gopalapuram, 600 086 Chennai, India; Department of Endocrinology & Metabolism, All India Institute of Medical Sciences (AIIMS), Ansari, New, Nagar, 110029 Delhi India

**Keywords:** Tobacco use, Southeast Asia, Secondhand smoke exposure, Population studies

## Abstract

**Background:**

Tobacco burdens in India and Pakistan require continued efforts to quantify tobacco use and its impacts. We examined the prevalence and sociodemographic and health-related correlates of tobacco use in Delhi, Chennai (India), and Karachi (Pakistan).

**Methods:**

Analysis of representative surveys of 11,260 participants (selected through multistage cluster random sampling; stratified by gender and age) in 2011 measured socio-demographics, tobacco use history, comorbid health conditions, and salivary cotinine. We used bivariate and multivariate regression analyses to examine factors associated with tobacco use.

**Results:**

Overall, 51.8 % were females, and 61.6 % were below the age of 45 years. Lifetime (ever) tobacco use prevalence (standardized for world population) was 45.0 %, 41.3 %, and 42.5 % among males, and 7.6 %, 8.5 %, and 19.7 % among females in Chennai, Delhi, and Karachi, respectively. Past 6 month tobacco use prevalence (standardized for world population) was 38.6 %, 36.1 %, and 39.1 % among males, and 7.3 %, 7.1 %, and 18.6 % among females in Chennai, Delhi, and Karachi, respectively. In multivariable regression analyses, residing in Delhi or Karachi versus Chennai; older age; lower education; earning less income; lower BMI; were each associated with tobacco use in both sexes. In addition, semi-skilled occupation versus not working and alcohol use were associated with tobacco use in males, and having newly diagnosed dyslipidemia was associated with lower odds of tobacco use among females. Mean salivary cotinine levels were higher among tobacco users versus nonusers (235.4; CI: 187.0-283.8 vs. 29.7; CI: 4.2, 55.2, respectively).

**Conclusion:**

High prevalence of tobacco use in the South Asian region, particularly among men, highlights the urgency to address this serious public health problem. Our analyses suggest targeted prevention and cessation interventions focused on lower socioeconomic groups may be particularly important.

**Electronic supplementary material:**

The online version of this article (doi:10.1186/s12889-015-1817-z) contains supplementary material, which is available to authorized users.

## Background

Tobacco use surveillance is critical, given that tobacco use, particularly cigarette smoking, is the leading preventable cause of mortality around the world [1]. It responsible for over 5 million deaths per year-more than HIV/AIDS, tuberculosis, and malaria combined [1,2]. Smoking increases the risk of cardiovascular disease (CVD) by 2 to 4 times, stroke by 2 to 4 times, and diabetes mellitus by 30-40 % [1]. In addition, smoking interacts with other CVD risk factors, such as hypertension and dyslipdemia, in increasing long-term CVD mortality [3–6]. Moreover, smoking causes diminished overall heath, including self-reported poor health, increased absenteeism from work, and increased health care utilization and cost [4].

Between 1980 and 2012, the global estimated age-standardized prevalence of daily tobacco smoking declined by 25 % for men and by 42 % for women; however, the population growth during this time contributed to a 41 % increase in male daily smokers and a 7 % increase for female smokers [7]. Moreover, over this period, cigarette consumption worldwide increased by 26 %, indicating continued growth of the global tobacco market [7]. According to the World Health Organization [WHO], if appropriate preventive measures are not taken, the number of annual deaths will increase to 10 million per year by 2030, with 70 % of them taking place in low-and middle-income countries [8]. Thus, continued efforts are needed to provide up-to-date estimates of tobacco use, including the broad range of tobacco products. Documenting correlates of use as well as the impact of these products on tobacco-related health conditions is critical for curbing the tobacco use pandemic and the related chronic health conditions, as it will help elucidate vulnerable populations and potential intervention targets.

A recent analysis of tobacco use data from nine countries in South and Southeast Asia found high prevalence of tobacco use in this region, ranging from 72 % in Indonesia to 32 % in Pakistan (with India’s prevalence being 34 %) [9]. This study also documented the use of tobacco in very diverse forms, particularly in India. Another study in India from 2009 to 2011 [10] found an overall tobacco use prevalence of 21 %, with 40 % of males being tobacco users compared to 5 % of females. A 2013 study in Pakistan [11] documented a current tobacco use prevalence (weighted to correspond to rural-urban population proportions in the sample) of 45 % among males and 6 % among females. In both India and Pakistan, some predictors of tobacco use include older age, male gender, low socioeconomic status, alcohol use, and rural geographic location [10,12,13]. This high prevalence is concerning given that South Asians have an increased risk of tobacco-related diseases, such as CVD and other CVD risk factors (e.g., diabetes, hypertension, and dyslipidemia) [14–17].

Given the public health importance, it is critical to track tobacco use within specific contexts in South Asia and characterize tobacco use patterns in terms of populations vulnerable to tobacco use and the types of tobacco being used. In this manuscript, we report the prevalence of tobacco use and its correlates among males and females in three specific cities in South Asia – Delhi, Chennai, and Karachi – using data from the Center for Cardio-metabolic Risk Reduction in South Asia (CARRS) Study. CARRS collects data regarding determinants (e.g., lifestyle factors) and occurrence of cardio-metabolic diseases; patient-reported quality of life; and costs using standardized tools and methods from representative samples in these three cities.

## Methods

### Study design

This study was approved by the Independent Ethics Committees of Public Health Foundation of India, All India Institute of Medical Sciences, Madras Diabetes Research Foundation and Aga Khan University, and Emory University’s Institutional Review Board. The CARRS Study [18] builds on the WHO STEPS model [19] to capture prevalence of risk factors, cardio-metabolic diseases (CMDs), and their socioeconomic impact. The study sites for the CARRS Study were metropolitan urban settings with large, heterogeneous populations (Chennai, population 4.68 million [20], Delhi, population 16.8 million [20], and Karachi, population 13 million [21]) that are growing due to continued births and migration from various parts of the country. The CARRS cohort study includes representative cross-sectional surveys of these three cities in South Asia as its baseline, conducted between October 2010 and December 2011 (see [18] for detailed methods).

### Participants

Households were selected in each of the three cities using multi-stage cluster random sampling techniques. Each city has its own distinctive municipal sub-divisions, encompassing municipal corporations, wards, and Census Enumeration Blocks (CEB), which were used sequentially as sampling frames to randomly select households. While wards were the primary sampling units (PSUs) for Chennai and Delhi, CEBs or clusters were the PSUs for Karachi. STATA version 10.1 (Statacorp, TX) and data from the most recent census (2001) were used to randomly select the wards, CEBs, and households. To give each household an equal chance of being selected for the study and to identify households constructed after the last census survey, manual listing and mapping of all households in each CEB was done before randomly selecting them.

Two participants, one male and one female, aged 20 years or older, were selected from each household. Those excluded from the study were pregnant women and bed-ridden individuals. Two methods were used for within household sampling. First, for households with one to two adults (≥20 years), the sampling strategy described in the 2002 Health Information National Trends Study (HINTS) in the USA was used [22]. According to HINTS, one or both individuals (one male, one female) were selected and enrolled into the study based on eligibility criteria and informed consent. Second, for households with more than two eligible adults, the “Kish method” used in the WHO’s STEPS surveys [23] was applied. There are two main steps in KISH method. First, all eligible participants from the household will be ranked according to age in decreasing orders (males followed by females). Participants are then selected using KISH table identifying the last digit of household and number of eligible participants [23]. Recruitment of participants and data collection were conducted through three visits to each participant’s place of residence, respectively. The sample size estimation, specifics on data collection, data management efforts, and quality control strategies have been published separately [18].

### Measures

For the current analyses, we included the variables listed below.

#### Correlates of tobacco Use

*Sociodemographic Factors*. Participants were asked to report age; gender; education level (up to primary, high/secondary, graduate, above graduation); occupation (not working, professional [e.g., doctor, lawyer, large business owner], trained [e.g., clerical, teacher, middle-level farmer], skilled [e.g., small business owner, skilled manual labourer, small farmer], semi-skilled [e.g., semi-skilled manual laborer, carpenter], unskilled [e.g., landless laborer, unskilled manual laborer); and income level per month (<10,000 Indian Rupees [INR] [<16,684 Pakistani Rupees [PKR] or < USD$165] vs. 10,000-20,000 INR [16,684-33,368 PKR or USD$165-330] vs. >20,000 INR [>33,368 PKR or > USD$330]).

*Health-Related Factors*. Prior research has documented lower body weight among current smokers than in never or ex-smokers [24,25]; thus, we measured body mass index (BMI; <18.0, 18-22.9, 23-24.99, > = 25) from height and weight. The association between alcohol use and tobacco use is also well documented [26,27]; thus, participants were asked, “How often do you use alcoholic beverages?” Those reporting using alcohol occasionally or regularly and those who quit using alcohol within the past six months were coded as current users (given the low alcohol use in these countries compared to western countries [28]); those reporting no use in the past six months or never use were reported as nonusers.

Self-report and biological verification diabetes, hypertension, and dyslipidemia were also included in the current analyses, given the association of these factors with smoking [3–6]. Biological sample collection involved drawing 15 ml of blood (in fasting state) and collecting urine (early morning void) from each participant. The samples were transported from field sites in cold chain to the laboratories for analysis. Sample aliquots were also stored in cryo-vials at - 80 degrees Celsius for future studies. The methods of analysis and external quality control have been standardized for all biological samples across the study sites.

Self-reported diabetes was defined as the participant reporting having diabetes (i.e., told by physician they have diabetes). Newly-diagnosed diabetes was defined as *not* having diagnosed diabetes but having FBG ≥ 126 mg/dl or A1c ≥ 6.5 %. Prediabetes was defined as FBG 100-125 mg/dl or HbA1c 5.7-6.5 %. If participants self-reported diabetes but had FBG < 126 mg/dl or A1c < 6.5 %, they still were considered to have self-reported diabetes, as treatment or management may have altered FBG. Self-reported hypertension was defined as the participants reporting having hypertension (i.e., told by physician they have hypertension). Newly-diagnosed hypertension was defined as *not* reporting hypertension but having SBP ≥ 140 or DBP ≥ 90 mmHg. Pre-hypertension was defined as SBP 120-139 or DBP 80-89 mmHg. Self-reported dyslipidemia was defined as the participant reporting having high lipids (i.e., told by physician they have high lipids). Newly-diagnosed dyslipidemia was defined as the participant reporting *not* having high lipids but having TC ≥ 200 mg/dl or LDL ≥ 130 mg/dl. If participants self-reported hypertension but had SBP < 140 or DBP < 90 mmHg, they still were considered to have self-reported hypertension, as treatment or management may have altered these parameters.

#### Tobacco exposure outcomes

*Tobacco Use History*. To assess tobacco use history, participants were asked, “Have you ever used tobacco in any form (smoking, chewing, snuff, etc.)?” and “In what forms have you consumed tobacco: In a smoking form? In a chewed form? In any other form (snuff, toothpaste etc)?” Participants were also asked, “At what age did you first start smoking regularly?” and “At what age did you first start consuming smokeless tobacco product regularly?”

*Current Tobacco Use*. Participants were asked, “Within the past 6 months, do you currently consume tobacco: regularly (once a week); occasionally (<once a week) or not at all?” Users were defined as those using regularly or occasionally. Current users were asked, “How often do you use: Smoking form? Chewed form? Any other form?” with the same response options (i.e., regularly (once a week); occasionally (<once a week) or not at all). They were also asked to report on level of current and past use of each of the following: tobacco smoking options (cigarettes; beedis; cigars; hukka/chelum/pipe); tobacco chewing options (pan with zarda; pan masala with zarda; guthka); or other forms (snuff; others).

*Secondhand Smoke (SHS) Exposure*. Participants were asked, “Are you exposed to tobacco smoke from others regularly (e.g. at home, at workplace regularly, while travelling, any other place)? How many days a week? How much time during a day?” Those reporting at least once a day in a week were coded as regularly exposed.

*Biomarkers of Tobacco Exposure*. Saliva samples were collected from 191, 214, and 196 randomly selected participants in Chennai, Delhi, and Karachi, respectively, to biochemically verify self-reported tobacco use. This tool was chosen due to greater acceptability of non-invasive, non-stimulated salivary sampling [29] and the high sensitivity of salivary cotinine in distinguishing active tobacco use from passive smoking with lower discrepancy between reported and measured prevalence as compared with urine or blood [30]. Also, Enzyme Immuno Assay (EIA) cotinine results have shown near perfect agreement with the reference standard GC/Mass Spectrometric confirmation [31]. Approximately 1000-2000 μL of saliva was collected in salivettes manufactured by SARSTEDT AG & Co., Germany. The samples were transported from field sites, in Cold chain, to the laboratories for analysis. Quantitative estimation of cotinine was performed by Enzyme immunoassay using kits from Salimetrics, PA 16803 USA. Samples with higher cotinine levels were re-estimated after further dilutions. The methods of analysis and external quality control were standardized for all biological samples across the study sites.

### Data analysis

Stata 12.0 (College Station, Texas, USA) was used for analysis. We used the *svy* technique for all analysis to account for the complex survey design [32]. Before any of the survey estimation commands were used, the **svyset** command was used to specify the variables that describe the stratification, sampling weight, and primary sampling unit variables. The age and sex standardized prevalence (95 % CI) of ever and current tobacco use were calculated for the three cities using World Bank’s estimates for regional as well as world standard population for comparison of prevalence. Bivariate analyses were done to examine the relationship of sociodemographic and health-related factors to current tobacco use (outcome) among males and females, respectively. Multivariable logistic regression analysis was then used to identify independent correlates of tobacco use among males and females, respectively, forcing the variables of interest into the models. Because the relationship of age with proportion of current tobacco use was curvilinear, we included a quadratic term for age in multivariable analysis. A p-value <0.05 was considered statistically significant.

## Results

A total of 17,274 individuals in 10,002 households were approached in the three study sites (7,596 participants in Chennai, 5,420 in Delhi, 4,258 in Karachi). From these, a total of 16,288 participants (n = 7,760 men, n = 8,527 women) were recruited (the overall response rate was 94.3 % at the participant level; Chennai 90.9 %, n = 6,906; Delhi 98.9 %, n = 5,365; and Karachi 94.3 %, n = 4,017). Response rate for providing blood and urine were 81.8 % (N = 13,327/16,288) and 84.3 % (N = 13,737/16,288), respectively. The number of participants with sufficient data retained to be able to conduct the final multivariate model was 11,260 (n = 5,062 men; n = 6,198 women).

Overall, 51.8 % of the participants were female, 61.6 % were below the age of 45 years, 27.5 % were in the 45-60 years age group, and 10.9 % were >60 years of age. Additional file 1: Table S1 provides comparisons of the CARRS sample to the regional and world population in relation to age, indicating underrepresentation among the younger age groups in the CARRS sample. (Note: Comparisons to the city populations also suggested similar underrepresentation of the younger age groups).

Lifetime (ever) tobacco use prevalence (standardized for world population) was 45.0 %, 41.3 %, and 42.5 % among males, and 7.6 %, 8.5 %, and 19.7 % among females in Chennai, Delhi, and Karachi, respectively (Table 1). The age and sex standardized estimates using the regional population and world population are also presented (Table 1). Past 6 month tobacco use prevalence (standardized for world population) was 38.6 %, 36.1 %, and 39.1 % among males, and 7.3 %, 7.1 %, and 18.6 % among females in Chennai, Delhi, and Karachi, respectively (Table 2).

**Table 1:** Ever use of tobacco products in Chennai, Delhi, and Karachi

	Any tobacco				Tobacco smoking				Chew tobacco				Other tobacco			
	Males		Females		Males		Females^§^		Males		Females		Males^§^		Females^§^	
City	%	CI	%	CI	%	CI	%	CI	%	CI	%	CI	%	CI	%	CI
**Prevalence with 95 % CI** ^**^**^																
Chennai	45.4	[42.8,48.0]	3.4	[2.6,4.1]	33.4	[30.8,36.1]	0.1	[0.1,0.3]	16.5	[14.7,18.2]	2.0	[1.4,2.6]	1.0	[0.6,1.5]	1.4	[0.9,1.9]
Delhi	43.9	[39.1,48.7]	7.9	[5.9,9.9]	30.4	[27.1,33.7]	1.6	[0.8,2.4]	20.3	[16.4,24.2]	5.9	[4.6,7.2]	0.6	[0.3,0.8]	0.8	[0.3,1.3]
Karachi	44.7	[41.9,47.5]	13.7	[10.7,16.7]	22.6	[20.3,24.9]	1.7	[1.1,2.3]	22.9	[19.3,26.5]	11.5	[8.6,14.4]	5.4	[4.7,6.1]	0.6	[0.3,0.9]
Prevalence with 95 % CI (standardized using regional population)*																
Chennai	45.3	[41.7,48.8]	7.4	[5.4,9.3]	34.7	[31.3,38.1]	0.2	[0.2,0.6]	12.8	[10.7,14.9]	4.8	[3.0,6.6]	1.6	[0.7,2.5]	2.5	[1.6,3.5]
Delhi	41.6	[36.7,46.6]	8.5	[6.1,10.9]	31.3	[27.3,35.2]	2.2	[0.9,3.5]	16.3	[13.2,19.3]	6.4	[4.6,8.3]	0.5	[0.2,0.7]	0.7	[0.3,1.0]
Karachi	43.1	[40.2,46.0]	18.7	[15.3,22.2]	25.1	[22.5,27.7]	2.2	[1.0,3.5]	18.2	[15.4,21.0]	16.0	[12.9,19.1]	5.2	[4.5,6.0]	1.0	[0.3,1.6]
**Prevalence with 95 % CI (standardized using world population)** ^**†**^																
Chennai	45.0	[41.4,48.6]	7.6	[5.5,9.7]	34.6	[31.1,38.0	0.2	[0.2,0.5]	12.6	[10.4,14.8]	5.1	[3.1,7.0]	1.7	[0.7,2.6]	2.5	[1.6,3.5]
Delhi	41.3	[36.3,46.3]	8.5	[6.0,11.0]	31.1	[27.1,35.0]	2.3	[0.9,3.7]	16.0	[13.0,19.0]	6.5	[4.5,8.4]	0.4	[0.2,0.7]	0.7	[0.3,1.1]
Karachi	42.5	[39.5,45.5]	19.7	[16.1,23.2]	25.3	[22.4,28.1]	2.3	[0.9,3.8]	17.3	[14.5,20.0]	16.9	[13.7,20.1]	5.3	[4.4,6.1]	1.0	[0.3,1.7]

^^^Prevalence data and 95 % confidence intervals adjusting for sampling weights

*Prevalence data and 95 % confidence intervals are age- and sex-standardized to the regional population. (Regional projected population by World Bank, 2010: India's population for Delhi and Chennai and Pakistan's population for Karachi)

^†^Prevalence data and 95 % confidence intervals are age- and sex-standardized to the world’s population

^§^Estimates likely unreliable due to small number of subjects in this category

**Table 2:** Past 6 month tobacco use prevalence and SHS exposure in Chennai, Delhi, and Karachi (standardized using world population)

	Chennai, N = 6,906				Delhi, N = 5,364				Karachi, N = 4,017			
	Males		Females		Males		Females		Males		Females	
Variable	%	CI	%	CI	%	CI	%	CI	%	CI	%	CI
**Any current tobacco use**	38.6	[34.5, 42.7]	7.3	[5.3, 9.3]	36.1	[31.7, 40.5]	7.1	[5.0, 9.3]	39.1	[36.2, 42.1]	18.6	[14.8, 22.3]
**Current tobacco smoking**	28.9	[25.3, 32.5]	0.5	[0.0, 1.0]	27.0	[23.6, 30.4]	2.4	[1.0, 3.8]	23.8	[21.1, 26.5]	4.8	[3.0, 6.6]
Cigarettes	22.7	[19.3, 26.1]	ǂ	ǂ	12.2	[10.6, 13.8]	ǂ	ǂ	20.8	[18.3, 23.2]	1.4	[0.2, 2.6]
Beedis	8.2	[6.3, 10.0]	ǂ	ǂ	15.5	[12.3, 18.8]	1.8	[0.5, 3.1]	0.1	[0.1, 0.3]	ǂ	ǂ
Cigars	0.1	[0.0, 0.2]	ǂ	ǂ	0.1	[0.0, 0.2]	ǂ	ǂ	ǂ	ǂ	ǂ	ǂ
Hookah; Chelum; Pipe	0.1	[0.0, 0.1]	ǂ	ǂ	0.5	[0.1, 0.5]	0.1	[0.1, 0.2]	0.0	[0.0, 0.1]	0.1	[0.0, 0.1]
**Current chewed tobacco use**	13.1	[11.1, 15.0]	4.8	[3.0, 6.6]	15.3	[12.6, 17.9]	5.6	[3.8, 7.3]	19.9	[17.3, 22.6]	16.3	[13.0, 19.6]
Tobacco chew	8.3	[6.4, 10.2]	3.1	[1.9, 4.3]	9.0	[7.1, 10.9]	2.5	[1.5, 3.5]	2.7	[1.9, 3.4]	2.0	[0.5, 3.5]
Pan with zarda	0.9	[0.0, 1.8]	0.5	[0.1, 0.9]	1.4	[0.9, 1.9]	1.5	[0.5, 2.5]	7.9	[5.7, 10.0]	11.0	[8.0, 14.0]
Panmasala	0.3	[0.1, 0.6]	ǂ	ǂ	0.7	[0.3, 1.1]	0.6	[0.2, 0.9]	0.6	[0.2, 1.0]	1.0	[0.1, 1.9]
Gutka	1.1	[0.6, 1.5]	ǂ	ǂ	3.7	[2.8, 4.6]	0.6	[0.2, 1.1]	4.4	[3.1, 5.7]	1.0	[0.3, 1.7]
**Current other tobacco use**	4.4	[3.1, 5.7]	3.3	[2.2, 4.5]	2.2	[1.5, 2.9]	2.1	[1.2, 2.9]	10.8	[9.6, 12.0]	4.0	[2.7, 5.3]
Snuff	1.5	[0.6, 2.4]	2.2	[1.4, 3.1]	0.1	[0.0, 0.2]	ǂ	ǂ	5.3	[4.5, 6.0]	1.0	[0.2, 1.7]
Others	0.3	[0.0, 0.5]	0.1	[0.1, 0.2]	0.4	[0.2, 0.6]	1.1	[0.6, 1.6]	0.4	[0.1, 0.8]	0.1	[0.0, 0.1]
**Regular exposure to SHS**	17.7	[15.3, 20.2]	10.0	[8.2, 12.3]	14.6	[11.9, 17.3]	7.6	[5.3, 9.8]	31.3	[28.0, 34.6]	12.7	[10.1, 15.3]
Salivary Cotinine*	Mean	95%CI	Mean	95%CI	Mean	95%CI	Mean	95%CI	Mean	95%CI	Mean	95%CI
Current tobacco use	168.5	[92.4, 244.6]	62.0	[15.1, 108.9]	555.5	[391.0, 720.0]	306.3	[162.8, 449.9]	157.1	[118.0, 196.3]	164.0	[103.0, 225.0]
No current tobacco use	41.3	[6.8, 75.7]	26.0	[9.3, 42.8]	39.6	[15.6, 63.7]	27.0	[0.0, 57.7]	25.5	[5.2, 45.9]	18.8	[15.1, 32.5]
Nonusers with SHS	68.2	[7.3,143.7]	24.6	[3.4, 45.9]	263.4	[106.5, 420.4]	43.9	[22.6, 65.1]	104.7	[54.9, 154.4]	55.0	[20.9, 89.0]
Nonusers without SHS	58.1	[15.4, 100.8]	56.8	[9.6, 104.0]	182.6	[58.1, 307.2]	47.6	[12.1, 83.0]	76.7	[48.3, 105.2]	30.8	[12.4, 49.1]

*Using sampling weights

ǂ not reported due to insufficient data

Note: The total for Cigarettes, Beedis, Cigars, Hookah, Chelum, and Pipe may be less than Current tobacco smoking as some of the observations were missing for the type of current tobacco smoking. The total for Tobacco chew, Pan with zarda, Panmasala, and Gutka may be less than Current chewed tobacco use as some of the observations were missing for the type of current chewed tobacco use. The total for Snuff and Others may be less than Current other tobacco use as some of the observations were missing for the type of current other tobacco use

Average age of initiation of tobacco smoking among lifetime smokers was 23.4 (SD = 0.7), 24.9 (SD = 0.8), and 24.5 (SD = 1.2) years in Chennai, Delhi, and Karachi, respectively (not shown in Tables). Average age of initiation of smokeless tobacco among lifetime users was 29.5 (SD = 0.9), 31.8 (SD = 1.2), and 27.4 (SD = 1.6) years in Chennai, Delhi, and Karachi, respectively. Among males, the mean age at initiation of any smoked tobacco use was 24.9 (SD = 0.4), 25.4 (SD = 0.4), and 24.6 (SD = 0.5) years old in Chennai, Delhi, and Karachi, respectively. The mean age at initiation for any smokeless tobacco use among males was 30.4 (SD = 0.8), 29.1 (SD = 0.7), and 24.9 (SD = 0.8), respectively in Chennai, Delhi, and Karachi. Similarly for female participants, the mean age at initiation for any smoked tobacco use was 35.0 (SD = 0.1), 28.9 (SD = 1.7), and 28.6 (SD = 1.6) and that for any smokeless tobacco use was 34.3 (SD = 1.5), 32.7 (SD = 1.1), and 28.00 (SD = 0.9) in Chennai, Delhi, and Karachi, respectively. Data averaged across all three cities, shows that the total mean age at initiation was lower in males than females for both smoked and smokeless tobacco use (M = 25.0, SD = 0.3 vs. M = 28.8, SD = 1.1, respectively for smoked tobacco and M = 28.2, SD = 0.6 vs. M = 30.9, SD = 0.7, respectively for smokeless tobacco). Among both males and females, age of initiation of tobacco smoking versus smokeless tobacco use was younger except in Chennai, where the mean age of initiation of smokeless tobacco use was lower than that for the smoked form among females (M = 34.3, SD = 1.5 vs. M = 35, SD = 0.1, respectively).

Table 2 presents data of different types of tobacco use among males and females. Males in Chennai most commonly reported use of cigarettes in Chennai (22.7 %) and Karachi (20.8 %) and beedis (15.5 %) in Delhi. Females most commonly reported use of chewed tobacco in Chennai (3.1 %) and Dehli (2.5 %) and pan with zarda in Karachi (11.0 %). Table 2 also shows that mean salivary cotinine values (ng/mL) were significantly higher in current tobacco users as compared to participants who reported no current tobacco use. Interestingly, there were no differences in cotinine levels in relation to SHS exposure versus no exposure among participants who reported no tobacco use.

Bivariate correlates of male tobacco use included: age (p < 0.001); lower education qualifications (p < 0.001); occupation (p < 0.001); lower income (p < 0.001); lower BMI (p < 0.001); current alcohol use (p < 0.001); and status of diabetes (p < 0.001), hypertension (p < 0.001), and dyslipidemia (p = 0.001) (Table 3). City of residence was not significantly associated with tobacco usage (p = 0.454). Factors associated with tobacco use in females were similar to that in males except that tobacco use was more prevalent in older age groups and a lack of an association with dyslipidemia status. Also, among females, city of residence was a significant determinant of tobacco usage (p = 0.001), with the highest prevalence seen in Karachi.

**Table 3:** Bivariate analyses examining tobacco use among males and females in Chennai, Delhi, and Karachi

	Male tobacco users			Female tobacco users		
Variable	%	95 % CI	P value	%	95 % CI	P value
**City**			0.454			0.0001
Chennai	40.9	[38.3, 43.5]		3.2	[2.6, 4.1]	
Delhi	40.6	[35.9, 45.5]		7.0	[5.3, 9.1]	
Karachi	42.5	[39.5, 45.5]		13.1	[10.4, 16.3]	
**Age group**			0.0001			0.0001
20-24	29.1	[24.7, 34.0]		3.4	[2.1, 5.6]	
25-34	44.1	[40.4, 47.8]		3.4	[2.2, 5.2]	
35-44	46.1	[42.8, 49.5]		6.4	[4.9, 8.4]	
45-54	44.2	[40.3, 48.2]		9.3	[7.5, 11.5]	
55-64	36.9	[33.7, 40.2]		10.6	[8.6, 13.0]	
>65	28.5	[23.2, 34.3]		13.2	[9.7, 17.7]	
**Education**			0.0001			0.0001
Above graduation	19.8	[16.1, 24.2]		0.4	[0.0, 2.5]	
Graduate	22.5	[19.4, 25.9]		1.4	[0.8, 2.7]	
High school/Secondary	43.3	[40.8, 45.8]		4.0	[3.2, 5.0]	
No formal education/up to primary	57.7	[54.4, 61.1]		14.9	[13.1, 16.9]	
**Occupation**			0.0001			0.009
Not working	32.4	[28.7, 36.4]		7.0	[5.9, 8.2]	
Professional	25.9	[21.3, 31.0]		5.9	[1.9, 16.9]	
Trained	27.3	[24.1, 30.8]		2.0	[0.4, 9.5]	
Skilled	40.8	[37.4, 44.2]		4.7	[2.6, 8.2]	
Semi-skilled	51.3	[47.8, 54.7]		5.5	[3.0, 9.7]	
Unskilled	56.5	[52.6, 60.3]		9.3	[6.5, 13.1]	
**Income**			0.0001			0.0001
<10000 INR	46.4	[44.2, 48.7]		7.9	[6.7, 9.3]	
10000-20000 INR	33.5	[30.1, 37.1]		5.1	[3.8, 6.8]	
>20000 INR	21.8	[18.6, 25.2]		1.8	[1.1, 2.9]	
**BMI (Kg/m2)**			0.0001			0.0001
<18.0	62.5	[56.5, 68.2]		14.3	[8.7, 22.7]	
18-22.99	45.1	[41.9, 48.2]		7.1	[5.7, 8.9]	
23-24.99	37.5	[33.1, 42.0]		6.8	[5.2, 8.9]	
> = 25	34.6	[32.1, 37.2]		5.7	[4.7, 6.9]	
**Alcohol use**			0.0001			0.013
Noncurrent alcohol user	31.7	[29.5, 33.9]		6.9	[5.8, 8.0]	
Current alcohol user	62.4	[59.2, 65.5]		34.7	[8.3, 75.8]	
**Diabetes based on FPG & HbA1c values and hx of diabetes**			0.0001			0.056
No/prediabetes	41.6	[39.4, 43.8]		6.0	[4.9, 7.4]	
Known diabetes	33.7	[30.3, 37.3]		9.1	[6.5, 12.6]	
Newly diagnosed diabetes	37.2	[33.8, 40.7]		6.5	[4.9, 8.6]	
**Hypertension**			0.0001			0.001
No hypertension	42.5	[39.9, 45.1]		6.1	[5.0, 7.4]	
Known hypertension	31.2	[27.7, 34.9]		9.4	[7.6, 11.6]	
Newly diagnosed hypertension	42.9	[39.9, 45.9]		8.1	[6.5, 10.1]	
**Dyslipidemia**			0.001			0.218
No dyslipidemia	40.5	[38.4, 42.7]		6.1	[5.0, 7.5]	
Known dyslipidemia	24.0	[17.8, 31.5]		5.6	[3.1, 10.0]	
Newly diagnosed dyslipidemia	40.0	[37.2, 42.8]		7.3	[6.1, 8.7]	

Note: P-values yielded by Pearson Chi-square test

In the multivariate logistic regression analyses (Table 4), correlates of tobacco use among both males and females included residing in Karachi or Delhi versus Chennai; older age; no formal or up to primary education; earning less income; and lower BMI. Additional correlates among males were being a semi-skilled laborer versus not and current alcohol use, whereas additional correlates among females included professionals versus not working and lower odds of having a known diagnosis of dyslipidemia. (Note that we explored including BMI and income as continuous variables and found similar results.) Multiple imputation was done for missing data, and the model was rerun, demonstrating similar results.

**Table 4:** Multivariate regression predicting tobacco use among males and females in Chennai, Delhi, and Karachi

	Males			Females		
Variables	OR	CI	p value	OR	CI	p value
**Constant**						
**City**						
Chennai®	Ref			Ref		
Delhi	1.66	[1.31,2.10]	0.0001	2.85	[1.91,4.26]	0.0001
Karachi	2.11	[1.71,2.59]	0.0001	4.33	[3.01,6.24]	0.0001
**Age (centered at mean)**	1.01	[1.00,1.02]	0.030	1.05	[1.03,1.06]	0.0001
**Age squared**	0.99	[1.00,1.00]	0.0001	0.99	[1.00,1.00]	0.055
**Education**						
Above graduation®	Ref			Ref		
Graduate	1.03	[0.70,1.51]	0.873	2.80	[0.30,26.24]	0.364
High/Secondary	1.51	[1.04,2.19]	0.031	8.47	[1.03,69.46]	0.047
No formal education/up to primary	2.67	[1.72,4.15]	0.0001	20.97	[2.53,173.62]	0.005
**Occupation**						
Not working®	Ref			Ref		
Professional	1.01	[0.65,1.57]	0.961	3.58	[1.12,11.40]	0.031
Trained	0.83	[0.58,1.17]	0.279	0.71	[0.19,2.64]	0.603
Skilled	1.06	[0.80,1.41]	0.671	0.93	[0.38,2.24]	0.870
Semi-skilled	1.36	[1.05,1.77]	0.020	0.56	[0.25,1.24]	0.151
Unskilled	1.23	[0.91,1.65]	0.173	1.52	[0.93,2.49]	0.095
**Income**						
<10000 INR®	Ref			Ref		
10000-20000 INR	0.81	[0.65,1.00]	0.052	0.68	[0.46,1.00]	0.051
>20000 INR	0.49	[0.36,0.65]	0.0001	0.37	[0.19,0.71]	0.003
**BMI**						
<18.0 ®	Ref			Ref		
18-22.99	0.50	[0.38,0.67]	0.0001	0.48	[0.27,0.85]	0.013
23-24.99	0.37	[0.26,0.52]	0.0001	0.41	[0.22,0.77]	0.006
> = 25	0.35	[0.26,0.46]	0.0001	0.40	[0.22,0.71]	0.002
**Alcohol use**						
No®	Ref			Ref		
Yes	4.17	[3.52,4.94]	0.060	8.18	[0.91,73.55]	0.911
**Diabetes based on FPG & HbA1c values and hx of diabetes**						
No diabetes®	Ref			Ref		
Known diagnosis diabetes	1.02	[0.80,1.29]	0.904	1.40	[0.89,2.20]	0.140
Newly diagnosed diabetes	1.02	[0.83,1.24]	0.878	0.97	[0.63,1.50]	0.883
**Hypertension**						
Normotension®	Ref			Ref		
Known diagnosis hypertension	0.87	[0.69,1.09]	0.208	0.91	[0.65,1.28]	0.580
Newly diagnosed hypertension	0.90	[0.76,1.06]	0.204	0.89	[0.67,1.19]	0.435
**Dyslipidemia**						
No dyslipidemia®	Ref			Ref		
Known diagnosis dyslipidemia	0.71	[0.41,1.22]	0.212	0.39	[0.16,0.93]	0.034
Newly diagnosed dyslipidemia	1.09	[0.94,1.27]	0.227	1.09	[0.83,1.44]	0.518
**Adjusted Wald test for all parameters**	F(24,69) = 24.10, p < 0.0001			F(24,68) = 10.71, p < 0.0001		

## Discussion

We found a high prevalence of tobacco use among males in Chennai, Delhi, and Karachi, while the prevalence of tobacco use among females is much lower. Specifically, lifetime tobacco use ranged from 41.3 % in Delhi to 45.0 % in Chennai among males and from 7.6 % in Chennai to 19.7 % in Karachi among females. Past six month use ranged from 36.1 % in Delhi to 39.1 % in Karachi among males and from 7.1 % in Delhi to 18.6 % in Karachi among females. Our estimates are in line with previous studies [10,11,33,34]. However, compared to our estimates in Delhi and Chennai, the Global Adult Tobacco Survey (GATS) found a higher current tobacco use prevalence in India, specifically 34.6 % (47.9 % among males, 20.3 % among females) [35]. Additionally, our estimates of current tobacco use among males and females in Karachi were lower than previously documented in Pakistan [11]. These differences in estimates may be related to the fact that rural males (not included in this study) have been shown to have higher tobacco use [10,12,13] or to variation in measures used. For example, many studies including the GATS assess current tobacco use by asking if participants currently use tobacco daily, less than daily, or not at all without stating a time frame, whereas the current study asked participants to report whether they had used these products in the past six months, which is more specific. To put these data in a global context, a 2012 study of 16 countries participating in GATS [35] found that 48.6 % of men and 11.3 % of women were tobacco users, with 40.7 % of men and 5.0 % of women using a combustible tobacco product [35]. As such, the tobacco use prevalence in these three cities is lower for males than in the countries included in the GATS but more similar to the females included in the GATS surveys [35].

The current study also documented a wide range of tobacco products used in this population, which is similar to findings in prior research [9]. Among males, use of smoked tobacco was highest in Chennai and lowest in Karachi, whereas chew tobacco rates were highest in Karachi and lowest in Chennai. Use of smoked or chewed tobacco among females was highest in Karachi. The most common tobacco product used in Chennai and Karachi was cigarettes, whereas beedis were most commonly used in Delhi. Other common tobacco products included tobacco chew, particularly in Chennai and Delhi, and Pan with Zarda, particularly in Karachi. The reasons for the differences in tobacco products used in these cities need to be explored.

In multivariable regression, factors associated with tobacco use among each males and females included residing in Karachi or Delhi versus Chennai. The reasons for these city differences are unclear; however, these findings may be partially attributed to lower price of tobacco products in Pakistan in comparison with India [36]. An additional finding indicated that, while Karachi had the highest tobacco use prevalence among the three cities for both men and women, lifetime tobacco use among males across the three cities were quite similar, which may suggest lower tobacco prices in Pakistan encouraging experimentation with tobacco. In addition, older age, lower education, earning less income, and lower BMI were correlates of tobacco use among both males and females, which aligns with prior research [10,12,13]. Another correlate of tobacco use among males was alcohol use. This is in line with well-established research in other countries documenting the connection between other substance use, particularly alcohol use, and smoking, both in this region and outside this region [10,12,13,37,38]. For females, another correlate of tobacco use was lower odds of having newly diagnosed dyslipidemia, which has not been documented previously.

The sociodemographic factors associated with tobacco use among the genders reflected one very important difference – males who were semi-skilled laborers versus not working were at greater risk for being a tobacco user, whereas females who were professionals versus not working were at greater risk for use. This may reflect the global trend of the tobacco industry targeting females in developing countries to increase their total market, particularly by targeting females who are educated and in urban settings [39–41].

An important strength of the current study was validation of self-reported tobacco use by estimating salivary cotinine level in a random subsample. As expected, the mean cotinine levels were higher among current tobacco users compared to nonusers. However, average cotinine levels were higher among both users and nonusers than shown in prior research [42,43]. For example, a 2000 study of 222 tobacco users and 97 nonusers found that mean salivary cotinine was 166 ng/ml in tobacco users and 6.3 ng/ml in nonusers. This study indicated an optimal cut-off to discriminate users from nonusers is between 7 and 13 ng/ml. The nonusers in the CARRS sample had higher cotinine levels than this cut-off point (range 18.8 to 41.6). This prior study indicated that smoking status of significant others was associated with higher cotinine levels among nonusers [42]; perhaps the high prevalence of tobacco use in these three cities implies high levels of SHS exposure impacting the cotinine levels of nonusers. However, another interesting finding from our study is that cotinine levels were not different between nonusers exposed to SHS versus not exposed, which warrants further examination.

The current study has important implications for research and practice. In terms of research, this study suggests the need for more longitudinal research regarding correlates of tobacco use among individuals in India and Pakistan, given the relatively limited scope of factors included in this data set. There is specific interest in the relationship between tobacco use and comorbid conditions like hypertension, diabetes, and dyslipidemia which cannot be adequately explored in a cross-sectional analysis like ours. We will have the opportunity to explore this as our cohort study matures. In addition, the social norms, tobacco control policies, and potential exposure to tobacco marketing should be examined in these differing contexts to determine the impact of these sociocontextual factors that impact tobacco use initiation and maintenance among males and females. Regarding practice, policies involved in the Framework Convention on Tobacco Control, particularly those impacting the social norms of tobacco use (e.g., public smoke-free policies, regulation of tobacco advertising) and systems to aid in cessation, must be supported in order to influence tobacco use initiation and maintenance among this population. In terms of practice implications, it appears from our analysis that people with known risks for heart disease and stroke (e.g., diabetes, hypertension) are less likely to smoke. Again, more longitudinal exploration of these relationships will be helpful, but regardless, practitioners must continue to address tobacco use in the clinical setting, particularly among patients with medical comorbidities.

### Limitations

A limitation of the CARRS model is that the study setting is urban and does not include the larger rural population; this sample also is limited due to an underrepresentation of the younger age groups [20,44]. Second, because of the cross-sectional nature of this study, we cannot determine the directionality of the relationships documented. However, the longitudinal nature of the CARRS study will allow us to address this limitation in future research. Moreover, the multivariate model allows us to determine the amount of variance in tobacco use accounted for by the other factors assessed in this study. Another limitation is that, because the primary aim of this study was not to explore all dimensions of tobacco use, several important factors potentially related to tobacco use (e.g., social norms, exposure to marketing) were not assessed. Additionally, our time-frame for tobacco use (i.e., past 6 month use) is somewhat unconventional but was used to capture the relatively low overall use of tobacco products among women in this region. This has implications for comparability to findings from other studies using other assessment approaches and other time frames. Finally, due to the multiple tobacco products assessed and the variability in number of products used, frequency of use, and variability in nicotine content across tobacco products, our current analysis of cotinine levels was mainly aimed at examining cotinine levels among current tobacco users versus nonusers, which confirmed differences between users and nonusers.

## Conclusions

Tobacco use prevalence is high, particularly among men, in Chennai, Delhi, and Karachi. Moreover, there is a broad range of tobacco products being used and differences in use prevalence of these products within these specific cities. Thus, tobacco control policy implementation is critical to address tobacco-related morbidity and mortality. Future research should examine psychosocial and contextual factors influencing tobacco use among those living in India and Pakistan. Specifically, factors impacting differential prevalence of tobacco use among males and females, the social norms of tobacco and other substance use, and the impact of health problems on cessation should be examined further. In addition, interventions and policies that might impact attitudes toward tobacco and social norms regarding tobacco use should be investigated and considered.

## Additional file

Additional file 1: Table S1. World and regional population versus CARRS population.

## Abbreviations

BMI: Body mass index
CARRS: Center for Cardiometabolic Risk Reduction in South Asia
CEB: Census enumeration blocks
CI: Confidence interval
CMD: Cardiometabolic diseases
CPD: Cigarettes per day
CVD: Cardiovascular disease
DBP: Diastolic blood pressure
FBG: Fasting blood glucose
FCTC: World Health Organization Framework Convention on Tobacco Control
HINTS: Health Information National Trends Study
HIV/AIDS: Human Immunodeficiency Virus/Acquired Immune Deficiency Syndrome
INR: Indian Rupees
LDL: Lowdensity lipoprotein cholesterol
M: Mean
PSU: Primary sampling units
PKR: Pakistani Rupees
SBP: Systolic blood pressure
SD: Standard deviation
SHS: Secondhand smoke
TC: Total cholesterol
USD: United States Dollars
