# Supplementary material for: A cross-sectional study of the prevalence and correlates of tobacco Use in Chennai, Delhi, and Karachi: data from the CARRS study
Source: BMC Public Health. 2015 May 11;15:483. doi: 10.1186/s12889-015-1817-z (PMC4432508; doi:10.1186/s12889-015-1817-z)
Supplement: Additional file 1: Table S1. — World and regional population versus CARRS population. [file 12889_2015_1817_MOESM1_ESM.docx]

**Table S1: World and regional population versus CARRS population**

|  | **World population*** | **CARRS population** | **Regional projected**  **population*** | | **CARRS**  **population** | |
| --- | --- | --- | --- | --- | --- | --- |
|  |  |  | **India** | **Pakistan** | **India** | **Pakistan** |
| **Males** | **N (%)** | **N (%)** | **N (%)** | **N (%)** | **N (%)** | **N (%)** |
| 20-24 | 3,08,376 (14.1) | 591 (7.6) | 59549 (15.9) | 9032 (19.2) | 359 (6.1) | 232 (12.3) |
| 25-34 | 5,35,072 (24.4) | 1614 (20.8) | 103617 (27.7) | 13623 (28.9) | 1229 (20.9) | 385 (20.4) |
| 35-44 | 4,76,899 (21.8) | 2128 (27.4) | 81123 (21.7) | 9414 (20.0) | 1677 (28.6) | 451 (23.8) |
| 45-54 | 3,75,813 (17.2) | 1723 (22.2) | 61796 (16.5) | 6800 (14.4) | 1365 (23.3) | 358 (18.9) |
| 55-64 | 2,65,642 (12.1) | 1026 (13.2) | 39932 (10.7) | 4251 (9.1) | 764 (13.0) | 262 (13.9) |
| 65+ | 2,29,942 (10.5) | 678 (8.7) | 28257 (7.6) | 3877 (8.2) | 474 (8.1) | 204 (10.8) |
| **Females** |  |  |  |  |  |  |
| 20-24 | 2,93,246 (13.2) | 588 (6.9) | 54724 (15.4) | 8779 (19.2) | 398 (6.2) | 190 (8.9) |
| 25-34 | 5,16,354 (23.3) | 2138 (25.1) | 95809 (27.0) | 13394 (29.3) | 1610 (25.2) | 528 (24.9) |
| 35-44 | 4,65,218 (21.0) | 2544 (29.83) | 75511 (21.3) | 9220 (20.2) | 1884 (29.4) | 660 (31.1) |
| 45-54 | 3,74,669 (16.9) | 1816 (21.3) | 58114 (16.4) | 6655 (14.5) | 1375 (21.5) | 441 (20.8) |
| 55-64 | 2,75,473 (12.4) | 979 (11.5) | 39206 (11.0) | 4113 (9.0) | 768 (12.0) | 211 (9.9) |
| 65+ | 2,91,343 (13.2) | 462 (5.4) | 32020 (9.0) | 3594 (7.9) | 367 (5.7) | 95 (4.5) |

*World standard population and regional projected population per World Bank 2010.
